# Supplementary material for: Infectivity, susceptibility, and risk factors associated with SARS-CoV-2 transmission under intensive contact tracing in Hunan, China
Source: Nat Commun. 2021 Mar 9;12:1533. doi: 10.1038/s41467-021-21710-6 (PMC7943579; doi:10.1038/s41467-021-21710-6)
Supplement: Supplementary file 2 — Reporting Summary [file 41467_2021_21710_MOESM2_ESM.pdf]

nature research

Corresponding author(s): Hongjie Yu

Last updated by author(s): Jan 20, 2021

Reporting Summary

Nature Research wishes to improve the reproducibility of the work that we publish. This form provides structure for consistency and transparency in reporting. For further information on Nature Research policies, see our Editorial Policies and the Editorial Policy Checklist.

Statistics

For all statistical analyses, confirm that the following items are present in the figure legend, table legend, main text, or Methods section.

n/a	Confirmed	
[ ]	[x]	The exact sample size (n) for each experimental group/condition, given as a discrete number and unit of measurement
[x]	[ ]	A statement on whether measurements were taken from distinct samples or whether the same sample was measured repeatedly
[ ]	[x]	The statistical test(s) used AND whether they are one- or two-sided<br>*Only common tests should be described solely by name; describe more complex techniques in the Methods section.*
[ ]	[x]	A description of all covariates tested
[ ]	[x]	A description of any assumptions or corrections, such as tests of normality and adjustment for multiple comparisons
[ ]	[x]	A full description of the statistical parameters including central tendency (e.g. means) or other basic estimates (e.g. regression coefficient) AND variation (e.g. standard deviation) or associated estimates of uncertainty (e.g. confidence intervals)
[ ]	[x]	For null hypothesis testing, the test statistic (e.g. F, t, r) with confidence intervals, effect sizes, degrees of freedom and P value noted<br>*Give P values as exact values whenever suitable.*
[x]	[ ]	For Bayesian analysis, information on the choice of priors and Markov chain Monte Carlo settings
[x]	[ ]	For hierarchical and complex designs, identification of the appropriate level for tests and full reporting of outcomes
[ ]	[x]	Estimates of effect sizes (e.g. Cohen's d, Pearson's r), indicating how they were calculated

Our web collection on statistics for biologists contains articles on many of the points above.

Software and code

Policy information about availability of computer code

Data collection	Microsoft Office Excel 2019 was used to collect data.
Data analysis	Statistical analyses were performed using the R software, version 3.6.3. The code, the synthetic dataset, and aggregated de-identified data to reproduce all the tables and figures in the main text and Supporting Information are publicly available.

For manuscripts utilizing custom algorithms or software that are central to the research but not yet described in published literature, software must be made available to editors and reviewers. We strongly encourage code deposition in a community repository (e.g. GitHub). See the Nature Research guidelines for submitting code & software for further information.

Data

Policy information about availability of data

All manuscripts must include a data availability statement. This statement should provide the following information, where applicable:

- Accession codes, unique identifiers, or web links for publicly available datasets
- A list of figures that have associated raw data
- A description of any restrictions on data availability

The data that support the findings of this study is available from the corresponding author upon reasonable request.

Field-specific reporting

Life sciences study design

All studies must disclose on these points even when the disclosure is negative.

Sample size	All of the 1,178 SARS-CoV-2 infectors and their 15,648 close contacts in Hunan Province were included in the study
Data exclusions	SARS-CoV-2 infectors with history of traveling to Wuhan and their contacts were excluded when analyzing factors associated with SARS-CoV-2 transmission.
Replication	No replication
Randomization	Observational study, no randomization
Blinding	Observational study, no blinding

Reporting for specific materials, systems and methods

We require information from authors about some types of materials, experimental systems and methods used in many studies. Here, indicate whether each material, system or method listed is relevant to your study. If you are not sure if a list item applies to your research, read the appropriate section before selecting a response.

Materials & experimental systems

n/a	Involved in the study	
☒	☐	Antibodies
☒	☐	Eukaryotic cell lines
☒	☐	Palaeontology and archaeology
☒	☐	Animals and other organisms
☐	☒	Human research participants
☒	☐	Clinical data
☒	☐	Dual use research of concern

Methods

n/a	Involved in the study	
☒	☐	ChIP-seq
☒	☐	Flow cytometry
☒	☐	MRI-based neuroimaging

Human research participants

Policy information about studies involving human research participants

Population characteristics	SARS-CoV-2 infectors and their contacts identified by Hunan SARS-CoV-2 surveillance system
Recruitment	All of the SARS-CoV-2 infectors and their close contacts in Hunan Province were included in the study. In order to avoid potential bias, SARS-CoV-2 infectors with history of traveling to Wuhan and their contacts were excluded when analyzing factors associated with SARS-CoV-2 transmission.
Ethics oversight	The collection of specimens, epidemiological and clinical data for SARS-CoV-2 infected individuals and their close contacts is a part of a continuing public health investigation of an emerging outbreak, ruled in the Protocol on the Prevention and Control of COVID-19 by the National Health Commission of the People's Republic of China, which was exempt from ethical approval (i.e., institutional review board assessment). However, staff of the local CDCs also informed the subjects that the collection of specimens would not pose any risk to personal privacy and information security before collecting specimens and took the specimens after obtaining verbal informed consents of the subjects. This study was approved by the ethic committee of the Hunan CDC with a waiver of informed consent for using the records of SARS-CoV-2 infected individuals and their contacts for scientific research. (IRB No. 2020005).

Note that full information on the approval of the study protocol must also be provided in the manuscript.
